# Embryonic heart rate is higher in species that experience greater nest predation risk during incubation

**DOI:** 10.1002/ece3.11460

**Published:** 2024-05-30

**Authors:** Alexander J. Di Giovanni, Todd M. Jones, Thomas J. Benson, Michael P. Ward

**Affiliations:** ^1^ Department of Natural Resources and Environmental Sciences University of Illinois at Urbana‐Champaign Urbana Illinois USA; ^2^ Illinois Natural History Survey, Prairie Research Institute University of Illinois at Urbana‐Champaign Champaign Illinois USA; ^3^ Smithsonian Conservation Biology Institute, Migratory Bird Center National Zoological Park Washington DC USA

**Keywords:** developmental rate, embryonic development, embryonic heart rate, incubation behavior, nest predation risk, reproductive success

## Abstract

Avian eggs develop outside of the female body, and therefore embryonic development is subject to multiple internal (physiological) and external (ecological) factors. Embryonic developmental rate has important consequences for survival. Within species, embryos that develop too quickly often experience deformities, disorders, or mortality, while embryos that develop slowly risk inviability and increase the time they are exposed to various sources of mortality in the nest. These contrasting forces may lead to interspecific variation in developmental rates. We investigated potential factors affecting embryonic heart rate (EHR), a proxy of development, across 14 passerine species in the field. More specifically, we investigated if nest predation risk, clutch size, seasonality, and egg volume influenced EHR. From previous research, we expected, and found, that EHR was positively associated with embryonic age and egg temperature. Species with greater nest predation risk had higher EHR, shorter incubation periods, and lower nest temperature variance. EHR increased as the season progressed and with egg volume, while EHR declined with clutch size. Bird species exhibit varying strategies to increase nestling and fledgling survival in response to predation risk, and these results suggest that variation in embryonic development may be related to species‐specific differences in nest predation risk.

## Graphical abstract

We investigated potential factors affecting embryonic heart rate (EHR), a proxy of development, in 14 wild songbird species in east‐central Illinois, USA. Within species we found that EHR was positively associated with embryonic age and egg temperature, while EHR among species were positively associated with nest predation risk. Consistent with past research on avian life histories, our findings suggest that predation risk plays an important role in governing variation in EHR (and developmental rates) among songbird species.

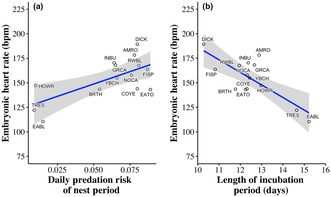

## 1 INTRODUCTION

Oviparity, external embryonic development within eggs, is common within a diverse suite of animal species. Research measuring embryonic development in oviparous species is critical to understanding the links between the external influences on development and the subsequent morphological, ecological, and fitness consequences (Du & Shine, 2015; Klinkhardt et al., 1987; Mirkovic & Rombough, 1998). As with other oviparous taxa, birds internally deposit all necessary resources for embryonic development within the egg with the exception of the gases that are exchanged through the porous shell. Altricial bird embryos are wholly reliant on parental incubation for their development and hatching success, making altricial birds ideal candidates for examining factors that influence embryonic development. Thus, unlike many reptiles that provide little or no post‐ovipositional care (Shine, 1988) or fish that are subject to ambient water temperatures (Perrone Jr & Zaret, 1979), birds actively incubate their eggs and are in greater control of embryonic development (Deeming, 2002).

In most bird species, females are the primary incubator mediating embryonic development by the timing, duration, and temperature at which they incubate the eggs (Stoleson & Beissinger, 1995). Avian embryos have an optimal thermal zone (35.5°–38.5°C; Webb, 1987) in which they develop (Martin et al., 2007; Nord & Nilsson, 2011). Exposure to temperatures below thermal optima for extended periods can decrease embryonic growth rates and lead to smaller nestlings or mortality due to limited internal yolk reserves and prolonged developmental periods (Olson et al., 2006; Ono et al., 1994; Ospina et al., 2018). Conversely, rapid developmental rates at temperatures above thermal optima can lead to deformities, disorders, or mortality and come at a cost of poor nestling condition after hatching and higher likelihood of nestling mortality (Leksrisompong et al., 2007; Ospina et al., 2018; Piestun et al., 2015).

While there may be an optimal temperature for embryonic development, other factors, such as nest predation risk, may select for rapid development to shorten the time young spend in the nest (Bosque & Bosque, 1995; Martin & Briskie, 2009; Merrill et al., 2021). Predation risk differs with nest type, where open‐cup nesting species experience greater predation risk than species that nest in cavities (Jones & Ward, 2020). As might be expected, species that experience lower nest predation risk (e.g., cavity‐nesting species; Merrill et al., 2021) tend to have longer incubation periods than species with higher nest predation risk (e.g., open‐cup nesters; Martin & Li, 1992; Ospina et al., 2018; Wesolowski & Tomialojc, 2005). A review of the growth rates of 64 passerine (altricial) birds found that nestling growth rates increased with nestling predation rates and that greater nestling predation risk was also associated with reduced feeding rates (Martin et al., 2011). Given broad interspecific variation in nestling growth rates, similar variation in embryonic developmental rates might be expected.

Besides predation risk, other factors such as clutch size, day of the year, and egg volume may impact embryonic development rates. Females may face trade‐offs between the number of eggs they lay and their ability to efficiently incubate all eggs within a clutch (Engstrand & Bryant, 2002; Thomson et al., 1998). Larger clutches require the female to expend more energy in incubation (Haftorn & Reinertsen, 1985; Thomson et al., 1998) and can be more difficult to effectively incubate (Engstrand & Bryant, 2002), leading to slower development. Additionally, the breeding season for most species is short (a few months), and juveniles in temperate locations must be prepared for harsh winter conditions or an energetically demanding migration by the end of the breeding season. Therefore, as the breeding season progresses, more rapid embryonic development may be needed for individuals from later‐initiated nests to develop enough to survive the winter or migration. Indeed, avian embryos are known to use external cues such as photoperiod to alter their developmental period (Clark & Reed, 2012; Cooper et al., 2011). Finally, females can vary their investment in eggs within and across clutches via the amount and composition of yolk, including but not limited to hormone concentrations (Hargitai et al., 2009; Lipar & Ketterson, 2000; Schwabl, 1997). Within species, egg size is a potential indicator of increased resource deposition (i.e., yolk and hormone inputs) and thus may be positively related to developmental rate of embryos (Christians, 2002; Krist, 2011; Williams, 1994).

A common approach for examining the influence of external factors on embryonic development is focusing on metabolic rate (Butler et al., 2004; Du et al., 2010; Kuroda et al., 1990; Sartori et al., 2015). However, oxygen consumption, which is often used as a proxy for metabolic energy expenditure, is also strongly correlated with embryonic heart rate (EHR; Butler et al., 2004; Du et al., 2010; Froget et al., 2001; Green, 2011), and EHR has frequently been used as a measurement of embryonic developmental rate (Du et al., 2010; Du & Shine, 2008; Pearson & Tazawa, 1999; Tazawa, 2005). Much of the past work on EHR has focused on incubation under artificial conditions (Khaliduzzaman et al., 2018; Lierz et al., 2006; Lourens et al., 2007; Noiva et al., 2014; Ono et al., 1994; Pearson & Tazawa, 1999; Tazawa, 2005). Artificially incubated eggs, however, are maintained at relatively stable conditions that may not capture the variability in female incubation behaviors that can negatively affect embryonic development in the nests of free‐living, wild birds (Olson et al., 2006; Ospina et al., 2018).

To better understand predictors of embryonic development in the nests of wild birds, we used a relatively new field technique—a non‐invasive ballistocardiogram— to sample EHR across a suite of 14 sympatrically breeding songbird species. We predicted that EHR would increase as the embryo ages and have a positive relationship with egg temperature, as has been found in other studies (Pearson & Tazawa, 1999; Sheldon & Griffith, 2018). Since predation risk is often correlated with the duration of a species' incubation period (Bosque & Bosque, 1995; Merrill et al., 2021), we predicted that along with shorter incubation periods species with greater predation risk would have faster developmental rates (have higher EHRs), reducing the amount of time spent in the nest and reduce their likelihood of predation, similar to developmental patterns observed in the nestling stage. Additionally, we expected that species would engage in more consistent nest attentiveness with less variation in egg temperatures when predation risk is high to increase the embryonic development rate. Due to increased internal resource investment in larger eggs, we predicted a positive relationship between EHR and egg volume. Finally, with a limited time for young to develop before migration or winter, we expected intraspecific EHR to increase across the breeding season.

## 2 METHODS

### 2.1 Study site, species, and monitoring

To examine embryonic development, we studied 14 coexisting passerine species at two study sites located in Vermilion County, Illinois, USA. Table 2 includes life history characteristics of each study species. The sites consisted of two‐ to 10‐hectare grassland patches in a forested matrix of primarily oak (*Quercus* spp.), maple (*Acer* spp.), and hickory (*Carya* spp.). From April to August 2019 and 2020, we searched for, located, and monitored open‐cup nests and ~125 nest boxes. We monitored each nest every 2–4 days once it was found and until the young fledged or the nest was depredated. We collected data from 1142 eggs from 388 nests across the 14 species over the two field seasons. Egg ages were determined by finding the nest at the laying stage or back‐calculating from the hatch date using known lengths of incubation for each species from previous work in the system (Jones & Ward, 2020). We followed the same procedures as Di Giovanni et al. (2023) to determine laying order, and thus egg ages, when we found nests early in the laying stage. Nests that were parasitized by the Brown‐headed Cowbird (*Molothrus ater*) were removed from the study because of potential effects on incubation behavior and clutch size (Robinson et al., 1995; Smith & Arcese, 1994).

We used a ballistocardiographic system, Buddy (Avitronics, Cornwall, UK), to measure EHR. To measure the cardiac contractions, the Buddy system measures the displacement of infrared light shined onto the egg caused by cardiac ballistic movement (i.e., the embryonic movements of blood pumping throughout their body), calculating EHR on a rolling average of four beats, updating every beat. The Buddy system does not come fully equipped for scientific measurements in the field and thus the units used in this study were modified by Avitronics to add nine‐pin serial port connectors for data collection via laptop and were adjusted to log EHR every 10 s. We used the program PuTTY (Tatham, UK) to collect and store the data received from each Buddy system on the connected laptop and used four Buddy monitors to simultaneously measure the EHR of each egg in each nest. We found no effect of egg orientation within the Buddy chambers on EHR using captive Zebra Finch (*Taeniopygia guttata*) eggs in the lab (A. J. Di Giovanni 2019 unpublished data). However, the eggs sampled in the field were all placed horizontally on the sensor cup for consistency. EHR was measured for 3 min at each nest check.

The Buddy system's estimates of EHR are unreliable in the early stages of embryonic development due to the lack of a developed cardiovascular system in the embryo (Pearson & Tazawa, 1999; Sartori et al., 2015). We therefore only collected EHR from embryos that had been incubated for at least 4 days. We excluded nests that had no egg age reference point (i.e., lay date or hatch date) from the analyses. EHR is linked to temperature, and we therefore used the maximum recorded EHR since the egg temperature can drop while the sampling occurs. Typically, the maximum EHR occurred within the first minute of sampling. Another potential issue with ballistocardiography is the influence of embryonic movement on EHR measurements (Pollard et al., 2016). The Buddy system's digital display includes an icon for embryonic movement, and our Buddy systems were modified by Avitronics to output an index of embryonic movement, an instantaneous waveform value ranging from 0 to 255. When eggs are relocated from the nest to the Buddy system, the embryo often moves for a short time period, so we omitted EHR readings using the following conditions: (1) EHR was 0 and there was no embryonic movement (i.e., the embryo ceased developing or very early in development), (2) there was extreme variation in EHR defined by a difference between maximum and mean EHR readings ≥225 bpm, suggesting inaccurate readings. We used this threshold because this extreme variation suggested heart rates fluctuating between <100 and >350 bpm, suggesting spurious data, (3) a mean movement index score ≥150, (4) an abnormally low EHR despite sufficient egg temperatures and embryo age (egg ≥ 5 days of incubation with heart rate of ≤100 bmp), suggesting an issue with embryo positioning within the egg, a potential abnormality with the eggshell, or instrument output error, or (5) eggs with damaged shells.

At each nest check, recorded eggshell temperatures (a reliable index of the internal egg temperatures; Leksrisompong et al., 2007) before and after EHR measurements using an infrared thermal camera (FLIR, model TG165). To measure egg volumes, we photographed each egg on a grided background using a Canon DSLR camera and analyzed the photos with the program ImageJ (Schneider et al., 2012), along with the egg measurement tool detailed by Troscianko (Troscianko, 2014). We placed iButton temperature loggers (Thermochron iButtons DS1921G, Maxim, San Jose, CA, USA) in 170 nests across nine different species to measure the nest temperature and characterize parental incubation behavior. The sample size for American Robin, Tree Swallow, Brown Thrasher, Eastern Towhee, and House Wren was too small to include in this analysis (*n* ≤ 9) and therefore not included. The iButtons were programmed to record temperature every 3 min and were placed in the nest on or after day four of incubation to reduce the likelihood of nest abandonment. From these iButton data, we were able to use temperature changes to determine when a female was in the nest and we calculated the percent of time the nest was incubated and the variance of nest temperatures. We extracted temperature data for the 3 h before taking EHR measurements.

### 2.2 Statistical analyses

We examined variation in EHR using generalized linear mixed models (package “lme4,” program R) using a Gaussian distribution. We examined the relationship between EHR and days after incubation initiation (embryo age), egg temperatures taken at arrival to the nest prior to EHR collection, week of season, number of eggs in the nest, and egg volume. Day of year and ambient temperature were highly correlated (*r* = .50, *p* < .001), and we retained day of year in our models because it is egg rather than ambient temperatures that are more important for the embryo. The slope of the relationship between embryo age and EHR was similar across species (based on overlapping confidence intervals in the interaction model), so we included additive effects of embryo age and species in all models of EHR. Clutch size varied due to partial predation events, nest parasitism, and dead eggs collected for another study (Di Giovanni et al., 2023) so we used the number of eggs at each nest check for our analyses. For each model, we included the identity of eggs, nests, and egg × nest as random effects to account for the non‐independence of multiple measurements per egg and multiple eggs per nest. Comparative studies often use phylogenetic corrections to control for perceived lack of statistical independence among species (Felsenstein, 1985; Pagel & Harvey, 1989). However, studies with low numbers of taxa (<20) often lack statistical power and produce inaccurate estimates of phylogenetic signal (Boettiger et al., 2012). As models only incorporated 14 species and our past attempts to control for phylogeny in the study system produced qualitatively identical results (Jones et al., 2020; Jones & Ward, 2020), we did not apply phylogenetic corrections in this case.

To explore differences in EHR with a variety of life history traits, we performed comparative analyses using the average EHR for each species after controlling for embryo age. We compared EHR with species‐specific average incubation period, average daily predation risk across the nest period, and average egg volume. We used nest type (open‐cup vs. cavity) to indirectly measure predation pressure. We used a logistic exposure method (SAS & Guide, 1990) to calculate daily predation risk of each species during the incubation period. We used Levene's tests to compare nest temperature variance across species. For these models, we used nest identity as a random effect to account for sampling the same nest multiple times.

## 3 RESULTS

**FIGURE 1:**
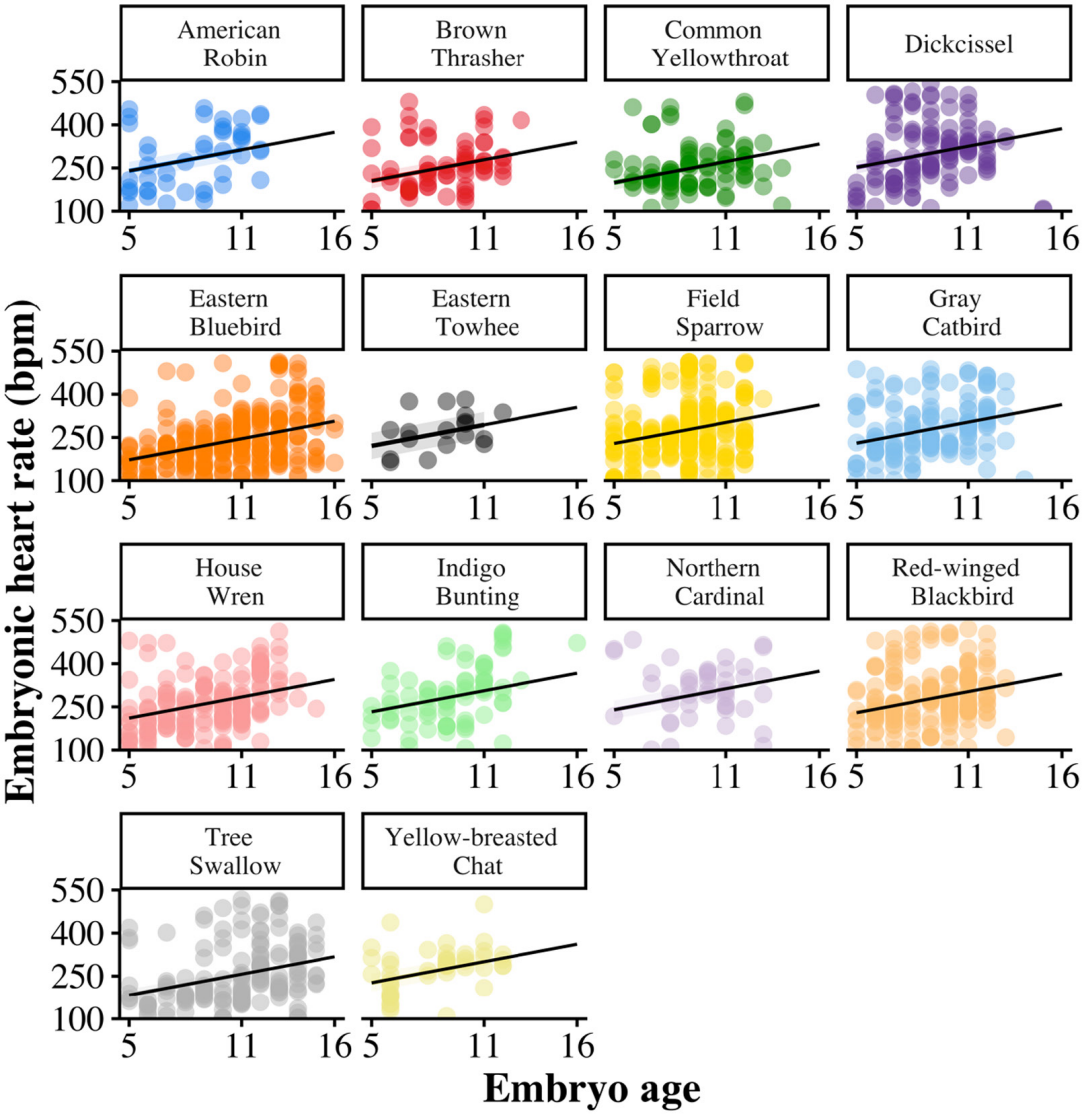
The increase of embryonic heart rate (EHR; beats per minute) across the incubation period of 14 species (*n* = 1142 eggs) of grassland and shrubland breeding songbirds in east‐central Illinois, USA, 2019–2020. Each measurement of EHR for every egg is represented as a single datapoint, see Table 2 for sample sizes. The black line represents the predicted average relationship between embryonic heart rate and egg age, as estimated by a linear regression model.

**FIGURE 2:**
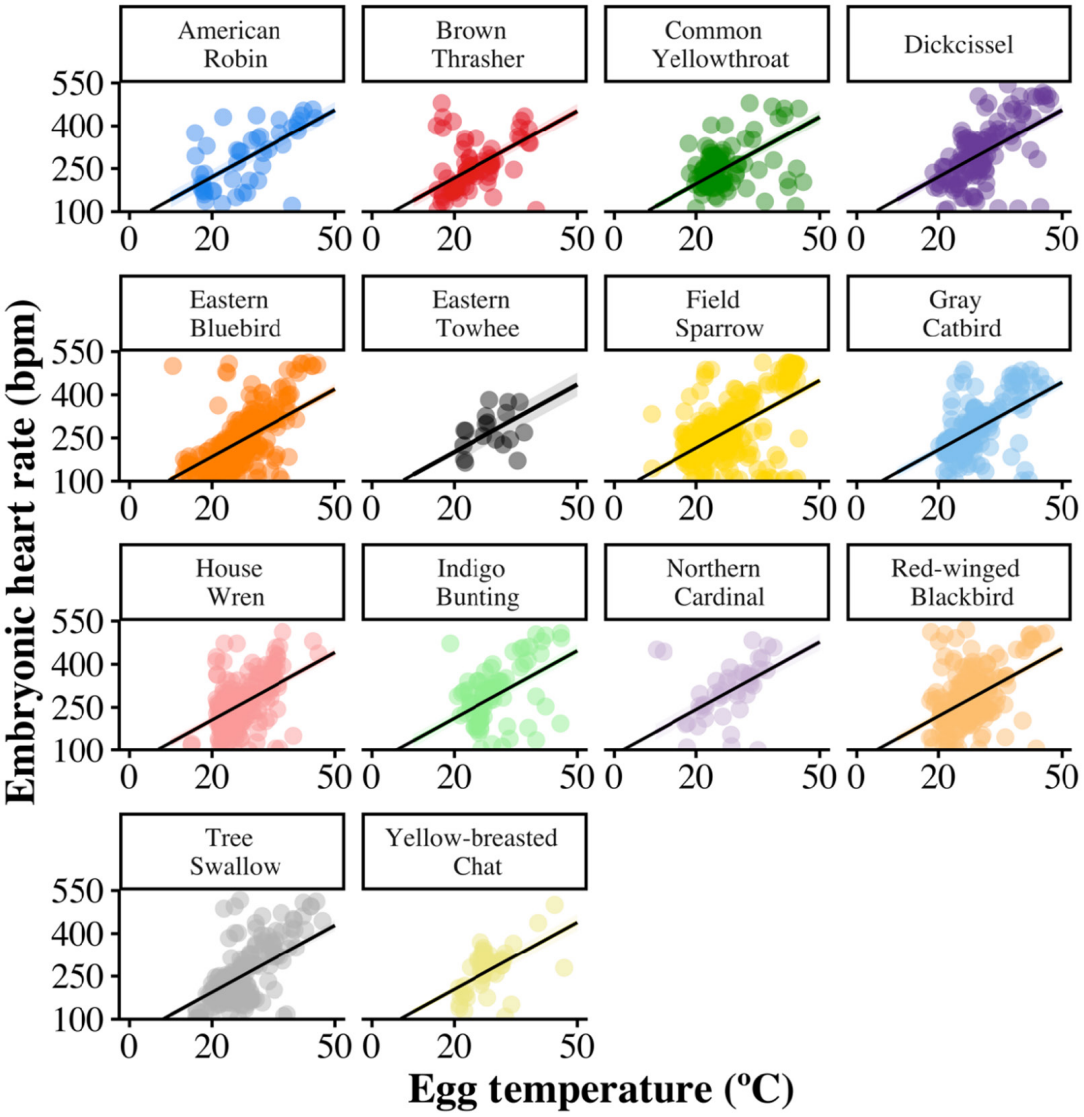
The increase of embryonic heart rate (EHR; beats per minute) with egg temperatures (°C) of 14 species (*n* = 1142 eggs) of grassland and shrubland breeding songbirds in east‐central Illinois, USA, 2019–2020. Each measurement of EHR for every egg is represented as a single datapoint, see Table 2 for sample sizes. The black line represents the predicted average relationship between embryonic heart rate and egg temperatures, as estimated by a linear regression model.

**FIGURE 3:**
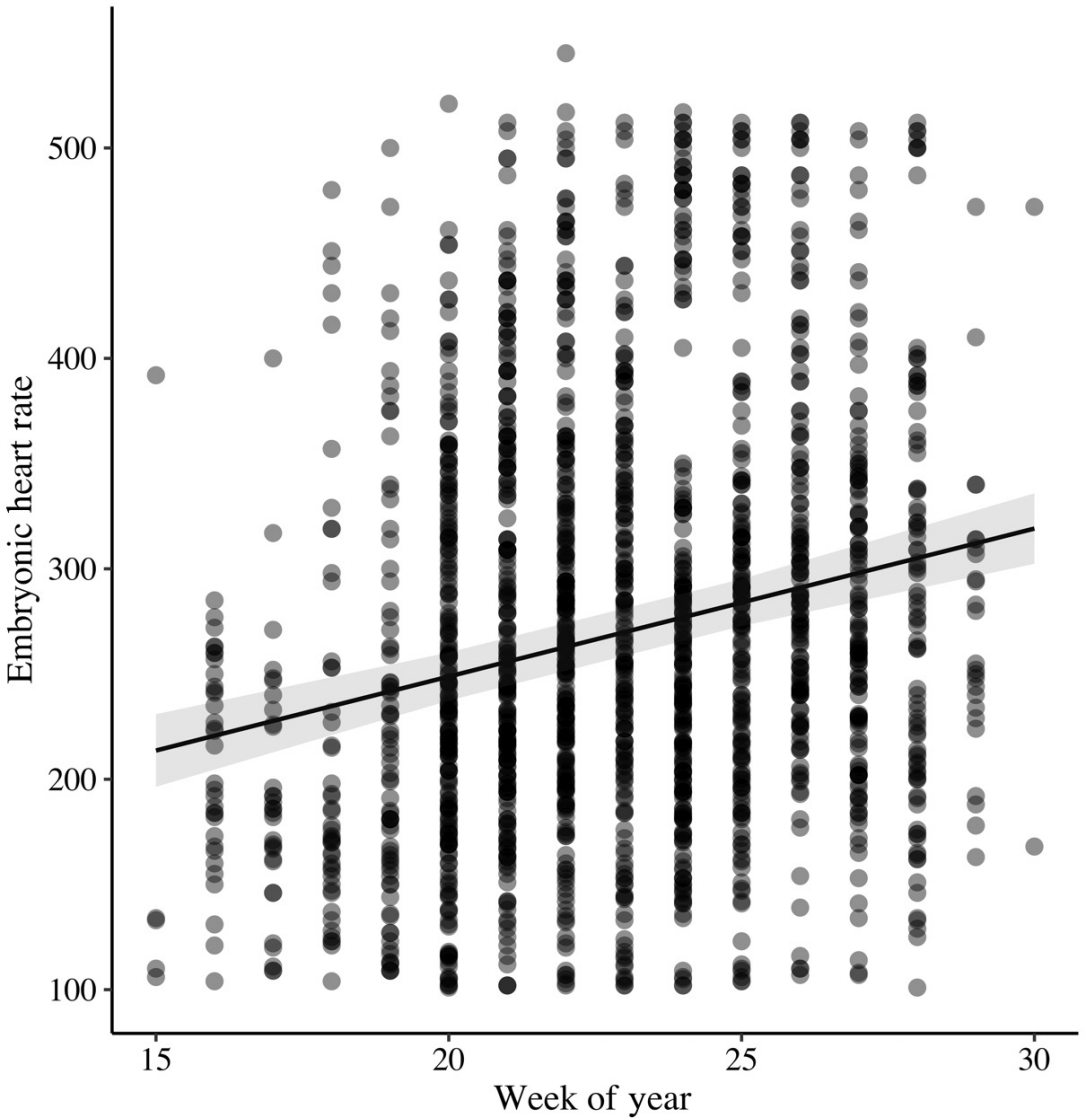
Embryonic heart rate (EHR; beats per minute) increases across the breeding season in 14 species of grassland and shrubland breeding songbirds in east‐central Illinois, USA, 2019–2020 after accounting for embryo age and controlling for species effects. Week 15 corresponds to the week of April 12, 2020. Each measurement of EHR for every egg is represented as a single datapoint, see Table 2 for sample sizes.

Across all species EHR increased with embryo age (*F*_1,1452_ = 175.39, *β* = 12.21 ± 0.92 [SE], *p* < .001; Figure 1; Table 1) and egg temperature (*F*_1,1611_ = 668.99, *β* = 7.92 ± 0.31 [SE], *p* < .001; Figure 2). EHR increased as the breeding season progressed (*F*_1,844_ = 59.37, *β* = 7.03 ± 0.91 [SE], *p* < .001; Figure 3). Additionally, EHR decreased with clutch size (*F*_1,1372_ = 8.40, *β* = −7.90 ± 2.73 [SE], *p* = .004) and increased with egg volume (*F*_1,948_ = 11.56, *β* = 23.94 ± 7.04 [SE], *p* < .001).

**TABLE 1:** Associations between model effects and predicting embryonic heart rate across the incubation period of 14 grassland/shrubland species (*n* = 1142 eggs within 388 nests) in eastern Illinois, USA, 2019–2020.

Effect	*β*	95% CI	*p*
Egg Age	12.2	10.4–14.0	<.001
Egg Temperature	7.9	7.2–8.4	<.001
Week of Year	7.0	5.3–8.8	<.001
Clutch Size	−7.9	−13.3 – −2.66	.004
Egg Volume	23.9	10.2–37.6	<.001

**FIGURE 4:**
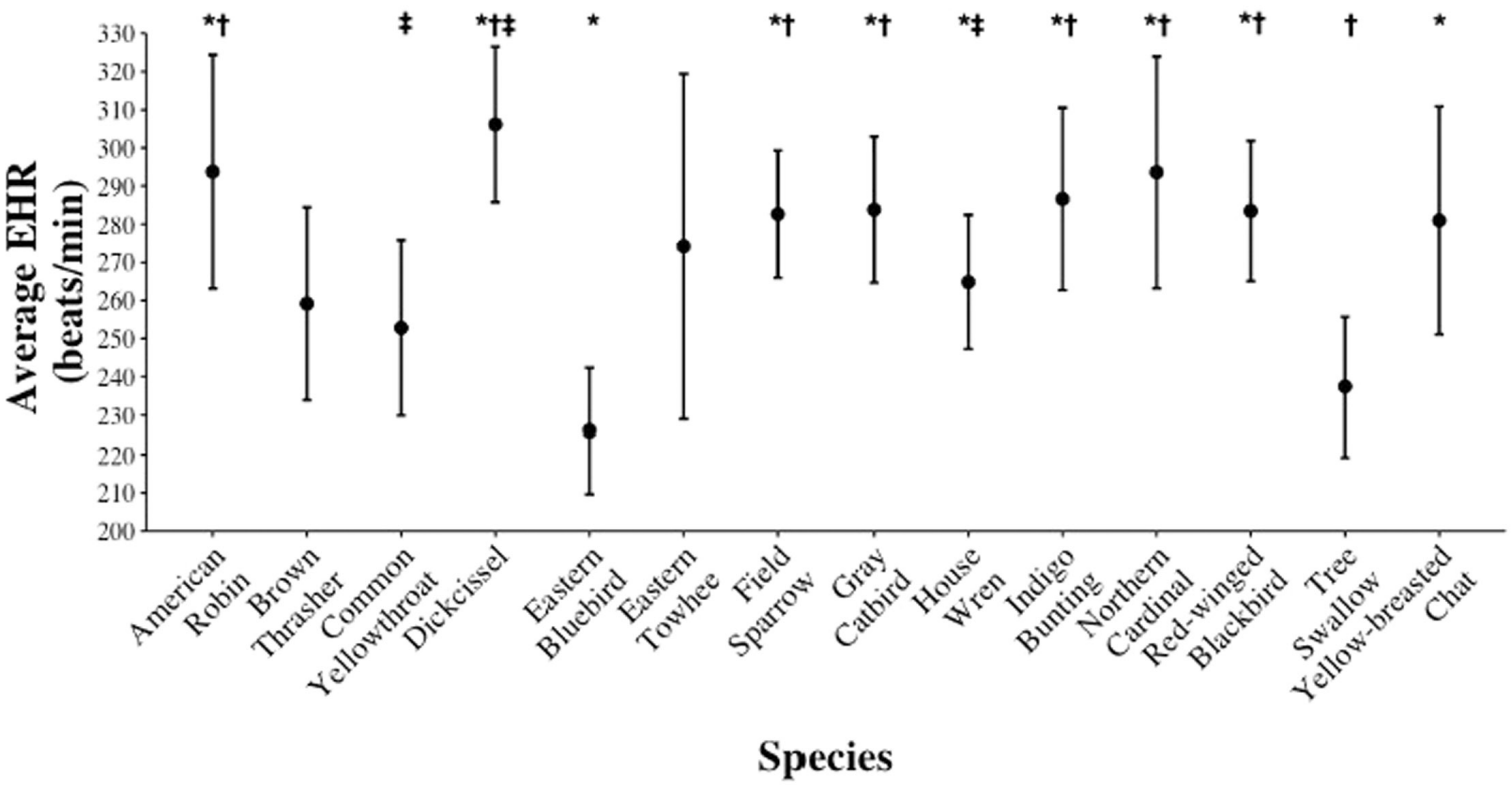
Embryonic heart rate (EHR; beat per minute) when controlling for embryo age (held at mean incubation period) across 14 species of grassland and shrubland breeding songbirds in east‐central Illinois, USA, 2019–2020. See Table 2 for sample sizes. Error bars represent 95% confidence intervals. Symbols above the boxplots indicate a statistically significant difference in average EHR between species.

**FIGURE 5:**
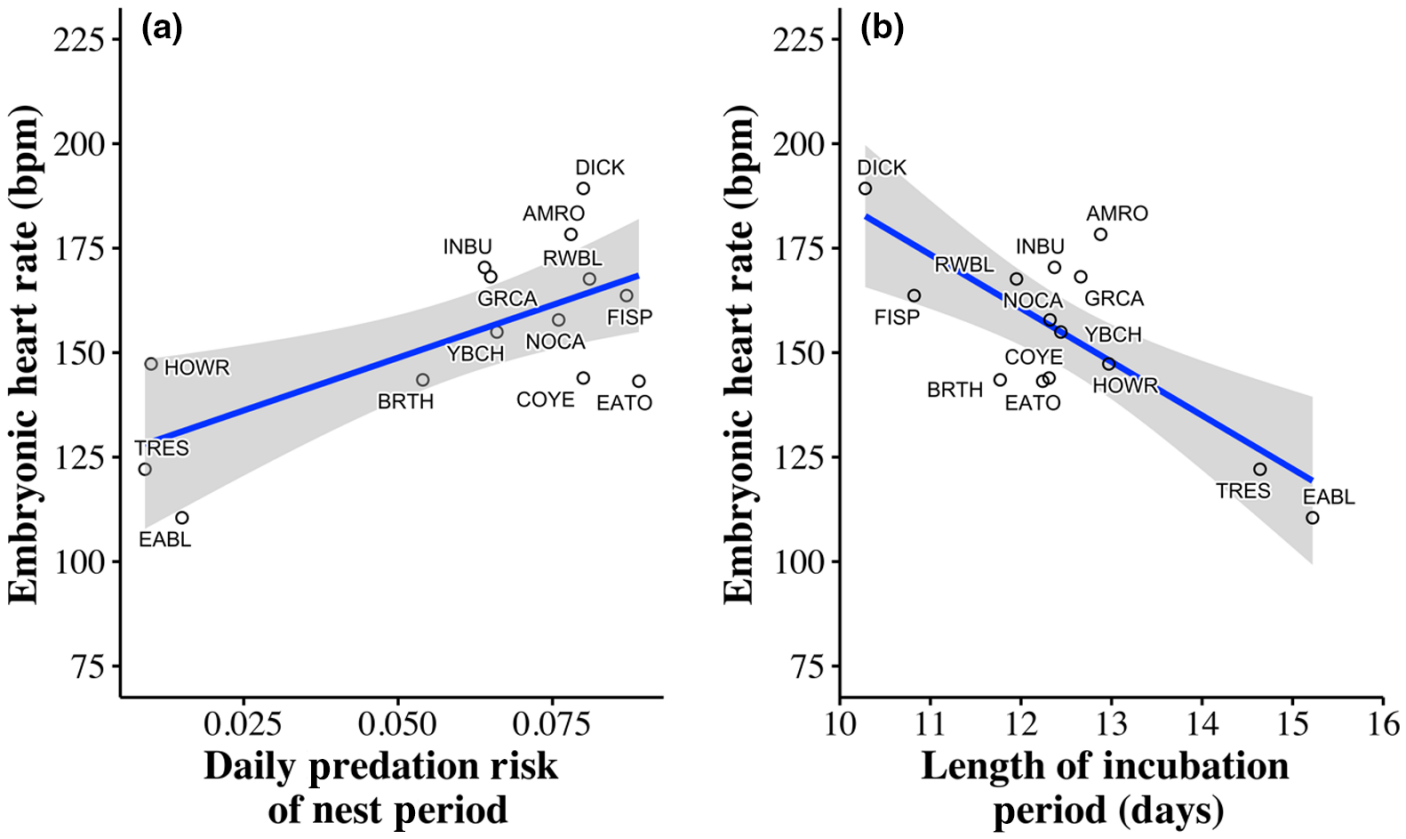
(a) The increase of average embryonic heart rate (EHR; beats per minute) with higher daily predation risk (percent probability of predation per day) in the nest period and (b) the decrease of average EHR with longer average incubation periods in 14 species of grassland and shrubland breeding songbirds in east‐central Illinois, USA, 2019–2020. Each species is represented as a single datapoint. The alpha code and sample sizes of each species can be found in Table 2. Shaded area represents standard error.

EHR differed among species (*F*_13,1051_ = 7.91, *p* < .001), with Eastern Bluebirds (*Sialis sialis*) and Tree Swallows (*Tachycineta bicolor*) having lower mean EHR than other species across embryo ages, and Dickcissels (*Spiza americana*) having higher mean EHR than other species (Figure 4). An individual species' mean EHR was positively associated with nest daily predation risk (*F*_1,12_ = 10.19, *β* = 504.55 ± 158.05 [SE], *p* = .008; Figure 5) and negatively related to the average length of the incubation period of the species (*F*_1,12_ = 17.28, *β* = −12.83 ± 3.09 [SE], *p* = .001; Figure 5).

**FIGURE 6:**
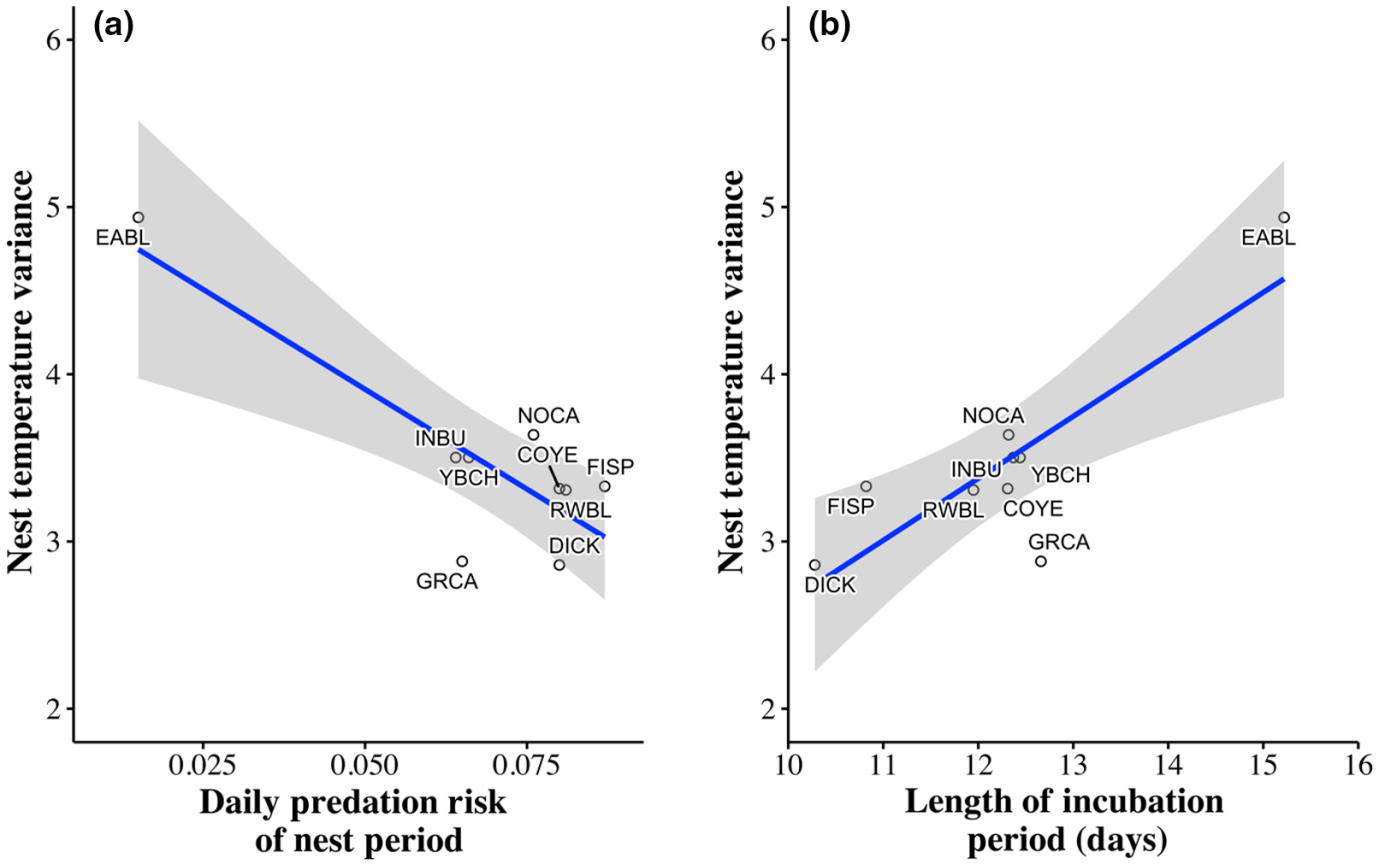
Nest temperature variance in 3 h prior to EHR measurement compared to (a) daily nest predation risk (percent probability of predation per day) and (b) the average length of the incubation period in days. Each species is represented as a single datapoint. The alpha code and sample sizes of each species can be found in Table 2.

There was a difference in nest temperature variance across the remaining species (*F*_8,161_ = 2.22, *p* = .03). These differences were driven by Eastern Bluebirds which had more variation in nest temperature than the other species. Additionally, Eastern Bluebirds spent the lowest percent of time in the nest and had the lowest mean nest temperature (Table 2). Species with higher EHR had lower nest temperature variance (*F*_1,7_ = 26.29, *β* = −0.02 ± 0.0048 [SE], *p* = .001). Nest temperature variance increased with daily nest predation risk (*F*_1,7_ = 17.44, *β* = −23.89 ± 5.72 [SE], *p* = .004; Figure 6) and incubation periods across species (*F*_1,7_ = 16.00, *β* = 0.37 ± 0.09 [SE], *p* = .005; Figure 6).

**TABLE 2:** Nest characteristics for 14 coexisting grassland and shrubland passerine species in east‐central Illinois, USA, 2019 and 2020.

Species	Scientific name	Species alpha code	Nest type	Average clutch size	Incubation period	Total nests EHR sampled	Total eggs EHR sampled	Percent of time spent incubating during daylight hours	Mean Nest temperature (°C)
Tree Swallow	*Tachycineta bicolor*	TRES	Cavity	5.2	14.6	23	98	—	—
House Wren	*Troglodytes aedon*	HOWR	Cavity	5.7	13.0	21	101	—	—
Gray Catbird	*Dumetella carolinensis*	GRCA	Open‐cup	3.7	12.7	34	105	69%	34.9
Brown Thrasher	*Toxostoma rufum*	BRTH	Open‐cup	4.0	11.8	17	51	—	—
American Robin	*Turdus migratorius*	AMRO	Open‐cup	3.4	12.9	13	29	—	—
Eastern Bluebird	*Sialia sialis*	EABL	Cavity	4.3	15.2	41	145	67%	32.9
Field Sparrow	*Spizella pusilla*	FISP	Open‐cup	3.4	10.8	71	185	71%	36.4
Eastern Towhee	*Pipilo erythrophthalmus*	EATO	Open‐cup	2.7	12.2	7	15	—	—
Yellow‐breasted Chat	*Icteria virens*	YBCH	Open‐cup	3.3	12.4	13	34	75%	36.3
Red‐winged Blackbird	*Agelaius phoeniceus*	RWBL	Open‐cup	3.4	12.0	48	123	69%	36.6
Common Yellowthroat	*Geothlypis trichas*	COYE	Open‐cup	3.2	12.3	19	64	75%	36.1
Northern Cardinal	*Cardinalis*	NOCA	Open‐cup	2.3	12.3	17	34	73%	35.0
Indigo Bunting	*Passerina cyanea*	INBU	Open‐cup	2.4	12.4	29	62	73%	35.8
Dickcissel	*Spiza americana*	DICK	Open‐cup	3.8	10.3	35	96	70%	37.0

## 4 DISCUSSION

Predation of dependent offspring is a critical source of natural selection helping to shape life history traits among animal taxa (sensu Martin & Briskie, 2009). In birds, the impact of nest predation on the length of the nestling period and associated nestling growth rates is well documented (e.g., Jones & Ward, 2020; Martin, 2015; Martin et al., 2018). However, the role of nest predation on embryonic growth has remained unclear, particularly in wild birds. Our results suggest that, as with the nestling stage, the risk of nest predation may impact embryonic development and incubation period lengths across songbird species. As indicated by EHR, species with higher risk of nest predation likely have higher rates of embryonic development, and consequently, have shorter incubation periods. Additionally, these findings integrate and expand on past findings regarding nest predation and juvenile metabolism (for which EHR is also a proxy), whereby nest predation is associated with nestling growth but not metabolism (Ton & Martin, 2020). Associations between nest predation and EHR therefore suggest that contributions of metabolism to juveniles may change between pre‐ and post‐natal stages, likely due to different roles of intrinsic (e.g., hormone) and extrinsic (e.g., food, parental care) factors across early life stages. Thus, findings from this research reinforce past studies on avian life histories (e.g., Martin, 2015; Martin & Briskie, 2009) by demonstrating that the important effects of nest predation on juvenile growth may extend to the embryonic stage, and how contributions of metabolism may vary across early life stages.

Consistent with avian life history theory, our findings suggest songbird species face key trade‐offs between predation risk and rates of development. While greater predation risk favors faster development to limit time spent in the nest (Martin, 2015; Martin et al., 2011; Remeŝ & Martin, 2002), slower embryonic development can produce higher‐quality young (Ricklefs, 1993). Indeed, cavity‐nesting species experienced lower predation pressure than open‐cup species (Wesolowski & Tomialojc, 2005) and had the longest average incubation periods of the species in this study. Cavity‐nesting species also had lower EHR than open‐cup nesting species when controlling for embryo age. Consequently, cavity‐nesting species may develop more slowly to produce higher‐quality young, as is indicated by their higher rates of post‐fledgling survival (Jones & Ward, 2020). Conversely, compared to cavity‐nesting species, open‐cup nesting species have relatively high predation risk and faster EHRs, so that their young may better escape predation pressure in the nest (Martin, 2015; Remeŝ & Martin, 2002). EHR can be increased by exposure to higher temperatures; however, there are substantial detriments to embryos when they develop too quickly, including malformities, diseases and disorders, and even mortality (Christensen, 2001; Leksrisompong et al., 2007; Molenaar et al., 2011; Piestun et al., 2015; Romanoff & Romanoff, 1972). In contrast, the primary threats to nest success are time‐dependent mortality risks, such as predation, and migrants are constrained to a breeding window that allows their young to develop sufficiently to perform migration. Consequently, these temporal constraints should be selected for rapid development in offspring (i.e., embryos; Remeŝ & Martin, 2002; Ricklefs, 1969, 1984). That the 11 open‐cup nesting species have similar EHRs and incubation periods (~10–12 days) but vary considerably in the duration of the nestling stage (~8–14 days difference) suggests there is strong selection pressure for rapid embryonic development in open‐cup nesting species, and that there are likely limits to hastening embryonic development.

As expected, and consistent with other research in altricial species (Pearson & Tazawa, 1999; Sheldon et al., 2018; Tazawa et al., 1994), we observed an increase in EHR with embryo age and egg temperature. Embryonic development is mediated by the parent and occurs at elevated temperatures that must be maintained by attending the nest. Therefore, EHR is positively associated with egg temperature, and this result is consistent with previous research (Deeming & Ferguson, 1991; Ono et al., 1994; Romanoff & Romanoff, 1972). We also found that EHR increased as the season progressed and was positively associated with egg volume. The seasonality in EHR may indicate that species are attempting to speed development under temporal constraints to fledge young more quickly. With migration following the breeding season for most of our study species, increased embryonic development as the season progresses may ensure that their offspring are developed enough to survive migration. The timing of breeding is a well‐studied aspect of avian life history, and many species attempt to breed in periods of ample resources for optimal nestling development (Cresswell & Mccleery, 2003; Lack, 1968; Martin, 1987). Parents can influence egg development with their investment, either externally through incubation behavior or internally through yolk, nutrient, or hormone levels (Schwabl et al., 2007). We did not have enough data to investigate changes in incubation patterns across the season; however, we did see an increase in egg volume as the season progressed (Di Giovanni, 2021) and that egg volume was positively correlated with EHR. Females have been observed to differentially allocate resources within their eggs as an adaptive strategy to their current environment, which affects their developmental speed (Merrill et al., 2019). Egg size has been linked to an increase in the total resources deposited in the egg and positively correlated with offspring growth (Eising & Groothuis, 2003; Krist, 2011; Williams, 1994). Clutch sizes tend to become smaller over the season which may lead to greater survival of nestlings (Lack, 1968). Furthermore, recent research provides evidence that embryos can sense external environmental cues and adjust their development accordingly (Clark & Reed, 2012; Du & Shine, 2015), which could account for some of the seasonal variations that we observed. While more research on the link between seasonal variation in development and both incubation behavior and egg volume is needed, this seasonal increase in EHR is consistent with broader patterns for increasing reproductive success.

We found that EHR varied with not only egg volume but the number of eggs in a clutch as well, with eggs in larger clutches exhibiting slower EHRs. Larger clutches have been shown to have more variable nest temperatures both within and among nests (Hope et al., 2021). Further, the heat generated via incubation is not as evenly distributed across all eggs in large clutches as compared to small clutches (Hope et al., 2018) which may impact EHR. While there is also evidence of larger clutch sizes not differing in hatching success from smaller clutches (Engstrand & Bryant, 2002; Smith, 1989), non‐optimal incubation temperatures are also known to subsequently affect nestling quality (Ospina et al., 2018). Additionally, even though we accounted for species identity in the analysis, the fact that cavity‐nesting species also have larger clutches leads to some ambiguity about which factor is more responsible for lower EHR. Further research is needed to more directly examine the relative influences of clutch size and nest type on both intra‐clutch variations in temperature and EHR.

Despite differences in life‐history traits, the species in our study displayed similar mean nest temperatures (Table 2). However, nest temperature variability differed among species, and temperature variation was related to EHR, incubation period length, and daily nest predation risk. Incubation is energetically costly to parents and may be just as costly as care during the nestling period (Nord & Williams, 2015). Consequently, parents face trade‐offs between the time devoted to incubation versus self‐maintenance (Boulton et al., 2010). Indeed, other studies that reduced the energetic costs of incubation demonstrated greater nest attentiveness (Ardia et al., 2009; Bryan & Bryant, 1999), suggesting it may not be energetically effective for females to increase EHR of their eggs any more than is required for the eggs to hatch successfully, especially if there is a cost to increasing embryonic development rates. Two of the cavity‐nesting species in our study, Eastern Bluebirds and Tree Swallows, have relatively long incubation periods. While we were unable to collect sufficient data on nest attendance for Tree Swallows, Eastern Bluebirds had lower nest attendance and lower mean nest temperatures than the open‐cup species we investigated. The reason for this lower attendance and nest temperature remains unclear, but the safer nest conditions may allow for greater self‐maintenance by the incubating female and a corresponding slower embryonic development, or the conditions with cavities may better buffer against extreme temperature fluctuations. Additionally, this may be due to differences in incubation patterns (i.e., partial incubation) and how time in the nest is distributed (e.g., see Austin et al., 2019; Wang & Beissinger, 2011), but more research is needed to examine incubation duration in the context of predation risk and the development of high‐quality young.

Predation risk is known to influence the development rate of nestlings (Martin, 2014), and we know from prior work that fledglings of some of the species with fast‐developing nestlings (e.g., Common Yellowthroats, Dickcissels, Field Sparrows) have higher predation rates than those with slower nestling developmental rates (cavity nesters; Jones & Ward, 2020). Even though we've observed variation in embryonic growth that is similarly associated with predation risk, the length of incubation periods tends to vary less than nestling‐period length, suggesting that species, especially those with higher likelihood of nest predation, may be pushing limits of embryonic development by minimizing variation of nest temperatures and elevating EHR to avoid nest predation. Future research should examine the limits of embryonic development (how quickly an embryo can develop to the point they can emerge from their egg) and the positive and negative aspects of development speed in both open‐cup and cavity‐nesting species.

## AUTHOR CONTRIBUTIONS

**Alexander J. Di Giovanni:** Conceptualization (equal); data curation (lead); formal analysis (lead); funding acquisition (supporting); writing – original draft (lead); writing – review and editing (lead). **Todd M. Jones:** Data curation (supporting); formal analysis (supporting); writing – review and editing (equal). **Thomas J. Benson:** Conceptualization (equal); formal analysis (supporting); writing – review and editing (equal). **Michael P. Ward:** Conceptualization (equal); funding acquisition (lead); writing – review and editing (equal).

## CONFLICT OF INTEREST STATEMENT

Authors declare no competing interests.

## Data Availability

Upon acceptance for publication, data will be uploaded to the Illinois Databank: https://databank.illinois.edu/
